# Comparison of three methods of extravascular lung water volume measurement in patients after cardiac surgery

**DOI:** 10.1186/cc7948

**Published:** 2009-07-06

**Authors:** Benjamin Maddison, Christopher Wolff, George Findlay, Peter Radermacher, Charles Hinds, Rupert M Pearse

**Affiliations:** 1 Barts and The London School of Medicine and Dentistry, Queen Mary's University of London, Royal London Hospital, London E1 1BB, UK; 2 Intensive Care Unit, University Hospital Wales, Heath Park, Cardiff CF14 4XW, UK; 3 Sektion Anästhesiologische Pathophysiologie und Verfahrensentwicklung, Universitätsklinikum, Ulm, Robert-Koch-Str. 8, 89081, Germany

## Abstract

**Introduction:**

Measurement of extravascular lung water (EVLW) by using the lithium-thermal (Li-thermal) and single-thermal indicator dilution methods was compared with the indocyanine green-thermal (ICG-thermal) method in humans.

**Methods:**

Single-center observational study involving patients undergoing cardiac surgery with cardiopulmonary bypass. Paired measurements were taken 1, 2, 4, and 6 hours after surgery. Bland-Altman analysis was used to calculate bias and limits of agreement. Data are presented as mean (SD) or median (IQR).

**Results:**

Seventeen patients were recruited (age, 69 years (54 to 87 years); Parsonnet score 10 (0 to 29)). Sixteen ICG-thermal measurements were excluded after blinded assessment because of poor-quality indicator dilution curves. EVLW volume as measured by the ICG-thermal technique was 4.6 (1.9) ml/kg, compared with 5.3 (1.4) ml/kg for the single-thermal method. Measurements taken with the Li-thermal method were clearly erroneous (-7.6 (7.4) ml/kg). In comparison with simultaneous measurements with the ICG-thermal method, single-thermal measurements had an acceptable degree of bias, but limits of agreement were poor (bias, -0.3 ml/kg (2.3)). Li-thermal measurements compared poorly with the ICG-thermal reference method (bias, 13.2 ml/kg (14.4)).

**Conclusions:**

The principal finding of this study was that the prototype Li-thermal method did not provide reliable measurements of EVLW volume when compared with the ICG-thermal reference technique. Although minimal bias was associated with the single-thermal method, limits of agreement were approximately 45% of the normal value of EVLW volume. The Li-thermal method performed very poorly because of the overestimation of mean indicator transit time by using an external lithium ion electrode. These findings suggest that the assessment of lung water content by lithium-indicator dilution is not sufficiently reliable for clinical use in individual patients.

## Introduction

Increased extravascular lung water (EVLW) volume during critical illness is associated with prolonged mechanical ventilation and increased mortality rates [1-4]. Quantification of EVLW volume may allow the use of therapeutic interventions to regulate lung water content, perhaps resulting in improved clinical outcomes [2,3]. Neither assessment of oxygenation nor chest radiography provides a reliable indication of EVLW volume [5-7]. No ideal method exists for measuring EVLW volume at the bedside.

In a previous laboratory study, we explored the use of indicator-dilution techniques to measure intrathoracic blood volume (ITBV) and EVLW volume [8]. The objective of this research was to develop a more convenient method of EVLW volume measurement by using lithium-thermal indicator dilution. Lithium chloride satisfies many of the criteria for an ideal indicator, including a good safety profile, small displacement volume, and minimal indicator loss [9-12]. However, in a recent laboratory investigation in porcine models of acute lung injury, both the existing indicator-dilution methods of EVLW volume measurement and our prototype Li-thermal method compared poorly with postmortem gravimetric measurements [8]. Given that each of these technologies was developed for use in humans, it is possible that measurements of EVLW volume would prove more reliable in humans. It is, therefore, necessary to compare indocyanine green-thermal indicator dilution, single-thermal indicator dilution, and the prototype lithium-thermal methods in humans. The aim of this study was to compare measurements of ITBV and EVLW volume made by using the indocyanine green-thermal (ICG-thermal), lithium-thermal (Li-thermal), and single-thermal indicator dilution techniques in patients after elective cardiac surgery with cardiopulmonary bypass.

## Materials and methods

This single-center, observational study was prospectively approved by the Local Research Ethics Committee. Patients undergoing elective cardiac surgery with cardiopulmonary bypass were eligible for recruitment. Perioperative changes in ITBV and EVLW volume in this population are significant and well described [13,14]. Written informed consent was sought before surgery. Exclusion criteria were refusal of consent, acute arrhythmias, significant cardiac valvular regurgitation, intra-aortic balloon counterpulsation, severe peripheral vascular disease, concurrent lithium therapy, pregnancy, and weight less than 40 kg. Anesthetic, cardiopulmonary bypass, blood transfusion, mechanical ventilation, and sedation practices were managed by clinical staff according to standardized local protocols. Paired measurements of ITBV and EVLW volume made by using each technique were taken 1, 2, 4, and 6 hours after surgery, as described in detail later. Initial plans for measurements at 24 hours were changed for pragmatic reasons, as detailed in the results. Indicator-dilution curves attained with each technique were analyzed in random order by CW, who was blinded to all other data. Curves were rejected if it was not possible to measure the relevant parameters manually.

### ICG-thermal measurement of ITBV and EVLW volume

The transpulmonary indicator-dilution technique allows the calculation of ITBV and EVLW volume according to Stewart's principle [15]. This describes the relation between cardiac output (CO), the volume throughout which an indicator distributes during transit (V), and the mean time taken for the indicator to pass from the point of injection to the point of detection (mean transit time, MTT) as follows:

As ICG remains confined to the vascular compartment, the distribution volume is equivalent to ITBV. The thermal indicator distributes throughout the thoracic water compartment, allowing measurement of intrathoracic water volume. EVLW volume may then be calculated by subtraction. ICG-thermal measurements were made by using the COLD-Z system (Pulsion Medical Systems, Munich, Germany) after central injection of iced 5% dextrose solution containing 0.2 mg/kg of ICG according to the manufacturer's instructions [16]. Arterial changes in temperature and ICG concentration were measured by using a thermistor-tipped spectrophotometric catheter inserted *via *an 18G femoral arterial catheter positioned with the tip at the level of the diaphragm (PV 2024 4FG; Pulsion Medical Systems).

### Li-thermal measurement of ITBV and EVLW volume

The principles underlying the Li-thermal method are the same as those of the ICG-thermal method. Li-thermal measurements were made by using the LiDCOplus system (LiDCO Ltd., Cambridge, UK) after central injection of 0.3 mmol (2 ml) of lithium chloride [17]. The arterial lithium ion concentration was measured by using an external lithium ion sensor attached to the radial arterial catheter via a 0.75-ml extension tube. Flow of arterial blood across the lithium sensor was regulated by using a battery-powered peristaltic pump. Time of injection was standardized through the use of a visual countdown on the monitor. The measured value of MTT includes lithium transit from the margin of the thorax to the external electrode. To allow calculation of the true physiologic value of MTT, we assumed a constant additional delay of 13.3 seconds. This value incorporates the known constant of 11.3 seconds for the indicator to transit the arterial catheter to the electrode (manufacturer's data), with published data from healthy volunteers suggesting that indicator transit from the margin of the thorax to the wrist would result in a delay of no more than 2.0 seconds [18]. Lithium cardiac output and lithium MTT were used to calculate ITBV. The Li-thermal calculation of EVLW volume was then made by using the measurement of intrathoracic thermal volume made by using the COLD-Z system at the same time point. EVLW volume was calculated from the lithium indicator-dilution measurement of cardiac output and MTT along with the thermal indicator value of MTT measured by using the COLD-Z system at the same time point.

### Single-thermal measurement of ITBV and EVLW volume

Single thermal indicator dilution allows the calculation of ITBV and EVLW volume solely from the thermal indicator dilution curve. Measurement of ITBV relies on the assumption of a fixed relation between ITBV and global end-diastolic volume (GEDV), as follows:

GEDV is calculated from measurements of total intrathoracic thermal volume and pulmonary thermal volumes, the latter being derived from analysis of the decay of the thermal indicator-dilution curve, applying Newman's hypothesis [15,19,20]. GEDV is obtained from the ICG indicator dilution curve to allow calculation of ITBV. EVLW volume is once again calculated by subtraction.

### Statistical analysis

A sample-size calculation was performed to ensure that the study had adequate statistical power to identify changes in ITBV during the postoperative period. Assuming a type I error rate of 5% and a type II error rate of 10%, we estimated that 20 patients would be required to detect a 1.5-ml/kg change in ITBV (SD, 4 ml/kg). Data are presented as mean (SD) where normally distributed and median (IQR) where not normally distributed. The comparison between paired measurements was tested by using the technique of Bland and Altman. Significance was set at *P *< 0.05.

## Results

Seventeen patients were recruited between July and September 2007. It was not possible to recruit 20 patients because of an insufficient number of spectrophotometric catheters. The baseline characteristics of these patients are presented in Table 1. Cardiorespiratory changes during the study period are presented in Table 2. After the recruitment of four patients, the final measurement time point was changed from 24 to 6 hours because of the clinical need to remove the femoral arterial catheter for postoperative mobilization. Before Bland-Altman analysis, 16 ICG measurements were excluded because of the poor quality of the indicator-dilution curve, leaving a total of 52 paired comparisons. All lithium dilution curves were of adequate quality.

**Table 1:** Baseline patient characteristics

Number	17
Age	69 (9) years
Gender	15 male
Weight	82 (19) kg
Parsonnet score	10 (8)
Duration of cardiopulmonary bypass	74 (17) minutes
Coronary artery bypass graft	13
Aortic valve replacement	2
Coronary artery bypass graft and aortic valve replacement as combined procedure	2

Data presented as mean (SD).

**Table 2:** Cardiorespiratory changes during study period. Data presented as mean (SD) or median [IQR]

Time	Hour 1	Hour 2	Hour 4	Hour 6
MAP (mm Hg)	71 (± 4)	71 (± 7)	79 (± 9)	75 (± 8)
CVP (mm Hg)	12 (± 4)	12 (± 3)	13 (± 4)	11 (± 3)
PaO_2 _(kPa)	14.8 [13.1–18.5]	15.3 [13.6–19.9]	15.2 [11.5–17.8]	13.7 [11.5–14.8]
PaO_2_:FiO_2 _(kPa)	34 (± 10)	37 (± 7)	36 (± 14)	34 (± 9)
Cumulative fluid balance (ml)	1,903 [1,740–2,631]	2,578 [1,969–2,908]	3,015 [2,294–3,379]	3,457 [2,621–4,506]

MAP = mean arterial pressure; CVP = central venous pressure.

**Figure 1:**
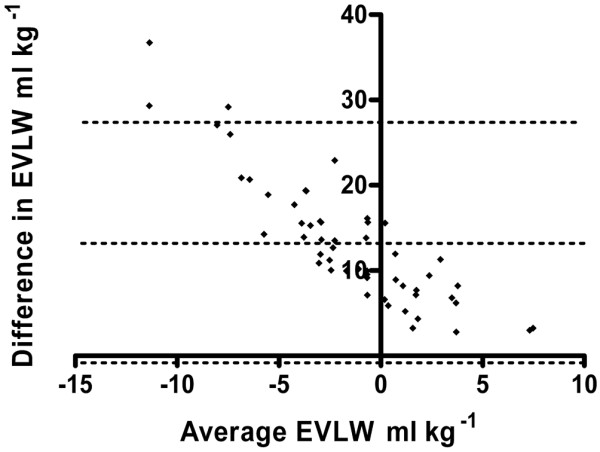
Bland-Altman analysis of paired measurements of extravascular lung water (EVLW) volume made by using the lithium-thermal indicator dilution method as compared with the indocyanine green-thermal indicator dilution method. Bias, 13.2 ml/kg; 95% limits of agreement, ± 14.4 ml/kg. Dotted lines indicate bias and limits of agreement.

**Figure 2:**
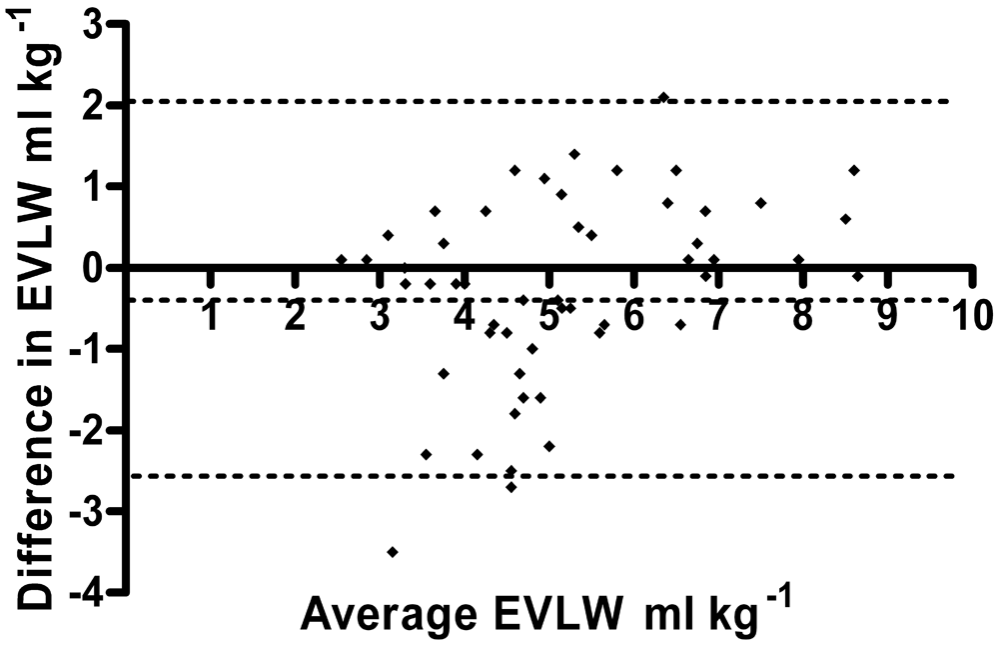
Bland-Altman analysis of paired measurements of extravascular lung water (EVLW) volume made by using the single-thermal indicator dilution method as compared with the indocyanine green-thermal indicator dilution method. Bias, -0.3 ml/kg; 95% limits of agreement, ± 2.3 ml/kg. Dotted lines indicate bias and limits of agreement.

EVLW volume as measured by the ICG-thermal technique was 4.6 (1.9) ml/kg, compared with 5.3 (1.4) ml/kg for the single-thermal method. Measurements taken with the Li-thermal method were clearly erroneous (-7.6 [7.4] ml/kg) and compared poorly with simultaneous measurements made by using the ICG-thermal method (bias, 13.2 (14.4) ml/kg) (Figure 1). For the single-thermal method, a more-acceptable bias was found, but limits of agreement remained poor (bias, -0.3 (2.3) ml/kg) (Figure 2). Agreement between the ICG-thermal and single-thermal methods in terms of percentage change in EVLW between time points also was poor (bias, 2.2% (72%)). ITBV and EVLW volume measurements at individual time points are presented in Table 3. Errors in the Li-thermal data resulted from a considerable overestimate of ITBV, due in turn to an overestimate of MTT (Table 4). Cardiac index, the other component variable of ITBV, was similar between the two techniques. As patients were rewarmed after cardiopulmonary bypass, the differences both in MTT and ITBV appeared to improve.

**Table 3:** Measurements of intrathoracic blood volume (ITBV) and extravascular lung water (EVLW) volume at individual time points by using three different methods of indicator dilution

Time	Hour 1	Hour 2	Hour 4	Hour 6
ICG-thermal ITBV (ml/m^2^)	794 (± 165)	856 (± 156)	880 (± 140)	915 (± 146)
Li-thermal ITBV (ml/m^2^)	1,271 (± 336)	1,318 (± 350)	1,309 (± 407)	1,203 (± 311)
Single-thermal ITBV (ml/m^2^)	777 (± 180)	827 (± 129)	880 (± 175)	880 (± 170)
ICG-thermal EVLW (ml/kg)	5.6 (± 2.1)	4.6 (± 1.9)	5.4 (± 2.0)	4.8 (± 1.4)
Li-thermal EVLW (ml/kg)	-7.8 (± 5.6)	-9.9 (± 5.6)	-7.9 (± 10.5)	-6.6 (± 7.0)
Single-thermal EVLW (ml/kg)	5.5 (± 1.7)	4.9 (± 1.4)	5.6 (± 1.4)	5.3 (± 1.0)

Data presented as mean (SD). Li-thermal = lithium-thermal indicator dilution; ICG-thermal = indocyanine green-thermal indicator dilution.

**Table 4:** Measurements of mean indicator transit time (MTT), cardiac index, and temperature at individual time points

Time	Hour 1	Hour 2	Hour 4	Hour 6
Li-thermal MTT (seconds)	35.1 (± 8.1)	33.2 (± 4.6)	29.4 (± 7.2)	28.7 (± 5.5)
ICG-thermal MTT (seconds)	19.0 (± 3.4)	18.5 (± 3.6)	17.8 (± 3.3)	18.1 (± 3.4)
Li-thermal cardiac index (L/min/m^2^)	2.3 (± 0.5)	2.4 (± 0.6)	2.6 (± 0.7)	2.6 (± 0.7)
ICG-thermal cardiac index (L/min/m^2^)	2.5 (± 0.7)	2.8 (± 0.6)	3.0 (± 0.6)	3.1 (± 0.6)
Core temperature (°C)	36.3 (± 0.5)	36.5 (± 0.6)	36.9 (± 0.5)	37.2 (± 0.5)
Peripheral temperature (°C)	30.6 (± 2.0)	31.6 (± 2.0)	32.8 (± 1.7)	33.1 (± 1.6)

Data presented as mean (SD). Li-thermal = lithium-thermal indicator dilution; ICG-thermal = indocyanine green-thermal indicator dilution.

## Discussion

The principal finding of this study was that the prototype Li-thermal method did not provide reliable measurements of EVLW volume when compared with the ICG-thermal reference technique. Whereas minimal bias was associated with the single-thermal method, limits of agreement were approximately 45% of the normal value of EVLW volume. The Li-thermal method performed very poorly because of the overestimation of mean indicator transit time by using an external lithium ion electrode. These data suggest that the Li-thermal method does not provide measurements of EVLW volume that are sufficiently reliable to guide clinical interventions in individual patients.

Previously we compared Li-thermal and ICG-thermal techniques with the gravimetric measurement of EVLW volume in a porcine model of acute lung injury. In this investigation, much closer agreement was found between the Li-thermal and ICG-thermal double-indicator methods [8]. However, in this previous investigation, the external lithium ion electrode was attached to a centrally placed femoral or carotid arterial catheter. These data suggest that, for accurate EVLW volume measurement by lithium indicator dilution, blood must be sampled *via *an arterial catheter sited within the aorta at the level of the diaphragm. ITBV is calculated as the product of cardiac output and MTT. Whereas measurements of cardiac output were similar for the two techniques, considerable differences were found in MTT. The assumption that the transit of lithium ions through the arterial circulation of the upper limb would be less than 2 seconds was incorrect. It is interesting to note that the difference between the Li-thermal and ICG-thermal measurements of MTT decreased as patients were rewarmed after cardiopulmonary bypass. Thus this source of error was not constant and cannot easily be adjusted for. Previous investigations have indicated that the loss of lithium ions from the vascular compartment during the measurement period does not affect the accuracy of cardiac-output measurement [10,11]. However, volumetric measurements may be more susceptible to this source of error, which also would result in an overestimation of MTT.

A study comparing EVLW volume measurement by using the ICG-thermal and single-thermal methods demonstrated inconsistencies between the two techniques [21]. In some cases, adjustment of the single-thermal algorithm is required to account for the individual circumstances of the experiment [22-24]. Similarly, in the current investigation, wide limits of agreement occurred between the single-thermal and ICG-thermal methods.

## Conclusions

In this study, the prototype Li-thermal indicator-dilution technique did not provide accurate measurements of EVLW volume. Along with those of our recent laboratory investigation [8], these findings suggest that accurate measurement of EVLW volume by lithium indicator dilution requires blood to be sampled from a central artery, *via *a catheter positioned with the tip at the level of the diaphragm.

## Key messages

• Increased extravascular lung water is an important clinical problem. Therapies to decrease lung water content may improve outcomes.

• The double-indicator dilution method of lung water measurement is considered the gold standard but is no longer commercially available.

• In this investigation, a prototype lithium-thermal method of lung water measurement performed poorly.

• The single-thermal indicator method was much more reliable than the prototype, but agreement with the double-indicator method was still disappointing.

## Abbreviations

CO: cardiac output; EVLW: extravascular lung water; GEDV: global end-diastolic volume; IQR: interquartile range; ICG: indocyanine green; ITBV: intrathoracic blood volume; Li: lithium; MTT: mean transit time.

## Competing interests

RP has received a research grant and equipment loans from LiDCO Ltd and honoraria for speaking from Pulsion Medical Systems.

## Authors' contributions

RP formulated the hypothesis and developed the protocol with CH. The investigation was performed by BM, at St. Bartholomew's Hospital, London, UK. CW, GF, and PR assisted in the data analysis. The manuscript was drafted by BM and RP. All authors approved the final version.
